# Eliciting broadly neutralizing antibodies against HIV-1 that target gp41 MPER

**DOI:** 10.1186/1742-4690-9-S2-P362

**Published:** 2012-09-13

**Authors:** D Han, H Habte, Y Qin, K Takamoto, C LaBranche, D Montefiori, MW Cho

**Affiliations:** 1 Albert Einstein College of Medicine, USA; 2 Duke University, USA; 3 Iowa State University, Ames, USA

## Background

The membrane-proximal external region (MPER) of HIV-1 gp41 is highly conserved and is targeted by broadly neutralizing antibodies (bnAbs). Thus, it is an attractive target for AIDS vaccine development. Here, we describe a mini-protein that can induce bnAbs in rabbits.

## Methods

We generated a mini-protein that is structurally rigid, yet efficiently recognized by 2F5, 4E10 and Z13e1. It contains the C-terminal 54 a.a. of gp41 ectodomain (gp41-54Q), which includes the HR2 and the MPER. A 6xHis tag at the C-terminus was used to attach gp41-54Q to Zn-chitosan, which served as an antigen carrier/adjuvant. Rabbits were immunized subcutaneously, 4 times, using two different schedules (wks 0, 4, 9 and 16 vs. 0, 8, 16 and 24). A total of 9 animals were immunized with gp41-54Q in 3 independent experiments. Antibody responses were evaluated by ELISA and in neutralization assays using both TZM-bl and A3R5.7 cells.

## Results

Eight of nine rabbits mounted bnAbs (89%). Neutralizing activity was observed against all but two of 44 viruses evaluated to date, including 27 Tier 2 viruses from clades A, B, C, D, AE, and CRF02_AG. Although Nabs could be detected after three immunizations, a fourth immunization was necessary for maximum neutralizing activity. The slower immunization regimen induced higher Nab titers, suggesting that longer rest periods improve affinity maturation. Neutralization inhibition analyses using various peptides identified one neutralizing epitope (N671, W672, F673 and D674) that overlaps with those recognized by Z13e1, 4E10 and 10E8 mAbs. Based on antibody absorption assays, there might be other non-linear epitopes. We are in the process of generating rabbit mAbs for more detailed analyses.

## Conclusion

We have successfully demonstrated that we can reproducibly induce bnAbs in rabbits using a mini-protein containing gp41 MPER. These results suggest that gp41 can be a promising vaccine immunogen.

